# Evaluation of a clinical pharmacist team-based telehealth intervention in a rural clinic setting: a pilot study of feasibility, organizational perceptions, and return on investment

**DOI:** 10.1186/s40814-020-00677-z

**Published:** 2020-09-10

**Authors:** Logan T. Murry, Christopher P. Parker, Rachel J. Finkelstein, Matthew Arnold, Korey Kennelty

**Affiliations:** 1 College of Pharmacy, The University of Iowa, 180 S. Grand Ave, Iowa City, IA 52242 USA; 2 Genesis Health System, 1345 West Central Park, Davenport, IA 52804 USA

**Keywords:** Telehealth, Team-based care, Diabetes, Rural, Pharmacists, Family medicine, Remote pharmacy service, Pilot, Return on investment

## Abstract

**Background:**

Remote, centralized clinical pharmacist services provided by board-certified clinical pharmacists have been shown to effectively assist in chronic disease management. We assess the feasibility of implementing a pharmacist-led, remote, centralized pharmacy service to improve A1c levels in patient with diabetes in a rural clinic setting.

**Methods:**

This was a non-randomized pilot and feasibility study. Participants were enrolled in a pharmacist-led telehealth intervention service, with data prior to enrollment used as baseline data for control. To be included, patients needed to have A1c readings of greater than 7% to be considered uncontrolled. A1c changes were reported for two groups based on A1c ranges: between 7 and 10% and ≥ 10%. Clinical pharmacists and clinical pharmacy interns initiated contact with patients via telephone communication and managed the patients remotely. The following outcomes were evaluated: organization perceptions (patients, providers, and clinic staff), changes in A1c, medication discrepancies, impact of an internally operated Patient Assistance Program, and potential return on investment (ROI).

**Results:**

Fifty-two patients were initially identified and referred to the service with 43 patients consenting to participate in the intervention. Patient and provider survey responses were recorded. In the initial analysis occurring during the first 3 to 5 months of the program, there was considerable improvement in diabetes control as measured by A1c. For patients with uncontrolled diabetes with a baseline A1c > 7% but less than < 10% and ≥ 10%, the intervention resulted in an A1c decrease of 0.57% and 2.55%, respectively. Clinical pharmacists and clinical pharmacy interns identified at least one medication discrepancy in 44% of patients, with number of discrepancies ranging from 1 to 5 per patient. At the conclusion of the study window, 42 potentially billable encounters were documented, which would have generated a net profit of $1140 USD, had they been submitted for reimbursement. Given the potential revenue generation, the service theoretically yields a ROI of 1.4 to 1.

**Conclusions:**

Initial results suggest that a pharmacist-led telehealth intervention has potential to decrease A1c levels in patients with diabetes, assist in identification of medication discrepancies, provide a positive return on investment for rural clinics, and potentially increase reimbursement for providers and clinics tasked with managing patients with uncontrolled diabetes.

## Key messages regarding feasibility

- What uncertainties existed regarding the feasibility?

The intervention team was uncertain how the clinical pharmacist intervention would be viewed by patients and provider groups in a rural primary care clinic setting. Furthermore, the intervention team was uncertain about what best practices would emerge for offering a telehealth intervention and maximizing patient benefits and outcomes in the rural clinic setting. Finally, it was unclear as to how the intervention may be sustained within the organization and what opportunity existed for billable events.

- What are the key feasibility findings?

Overall, the intervention was well received by patients and providers attributed to the rural clinic. Both groups appreciated the additional support provided by pharmacists, with some patients willing to pay for continued access to the intervention. Allowing clinical pharmacists expanded access and abilities in the EHR may be associated with improved intervention outcomes. Based on potential opportunities to bill for pharmacy services, the clinical pharmacy intervention has the ability to generate clinic revenue while improving patient outcomes and increasing clinic staff physician perceptions of additional time to see patients.

- What are the key implications of the feasibility findings for the design of the main study?

In order to maximize the intervention, the main study will set out to obtain extensive read and write capabilities for clinical pharmacists within participating site EHRs at the onset of the study period. This finding came from clinic provider recommendations and is supported in previous primary literature and reviews.

## Background

Over 30 million Americans have diabetes, a condition afflicting 9.4% of the US population [1]. In 2015, diabetes was the 7th leading cause of mortality, directly accounting for 79,535 deaths and contributing to another 252,806 deaths [2]. Additionally, the economic costs of diabetes were estimated to be nearly $327 billion in 2017 [3]. Despite the health and economic impact of the condition, effective implementation of evidence-based interventions that reduce diabetes-related morbidity and mortality remains a substantial challenge. Chronic diabetes management is particularly challenging in rural clinic settings, as patients in these clinics are more likely to have uncontrolled A1c values compared to their urban counterparts [4]. Given the differences in controlled A1c in rural and urban populations, additional care and resources are necessary to reduce disease burden and increase diabetes control for patients seeking care in rural clinic settings. The American Diabetes Association recommends using a team-based approach to optimize care for patients with diabetes [5]. Team-based care is the utilization of healthcare professionals from multiple disciplines to manage care for patients, with the goal of improving primary care quality and efficacy [6]. Team-based care has become a strategy to reduce gaps in therapy by using pharmacists to help manage chronic conditions and decrease clinical inertia [7–11].

Type II diabetes is a progressive disease, with disease management requiring multiple medications with frequent follow-up and monitoring. As rural clinics often do not have the resources to employ a full-time clinically trained pharmacist, this intervention provides patient access to pharmaceutical care. While multiple pharmacies exist in the community of interest, these pharmacies may be unable to provide clinical services due to workload and lack of clinically trained pharmacists. For these reasons, a telehealth intervention of clinical pharmacy services is a potential method to maximize health outcomes for rural populations, particularly for patients living with diabetes [11]. Through research grant funding, we have developed a telehealth clinical pharmacy service to support under-resourced primary care clinics with chronic disease state management [7, 12, 13]. Currently, the telehealth intervention is being refined and transitioning from research grant funding to a self-sustained model of remote, centralized clinical pharmacy services.

## Objectives

A pilot project was performed in 2017 within the context of sustaining the clinical service independent of research grant funding. The primary objective of this study was to determine workflow processes as well as patient, provider, and clinic staff experiences with the clinical pharmacy service. The secondary objectives were to evaluate the intervention’s impact on the following endpoints for the specific patient population recruited: change in A1c, medication discrepancies, patient cost savings, and estimated potential return on investment (ROI) to determine sustainability of providing this clinical service to rural primary care clinics.

## Methods

### Subjects and setting

This was a non-randomized pilot and feasibility study taking place in a small, rural community in Iowa, with the primary focus of the intervention being management of uncontrolled diabetes. The study used a non-equivalent group, pre-post design. The pilot intervention was initiated June 2017 and was completed November 2017, with patients enrolled on a rolling basis and followed for 6-month periods throughout the intervention period. The pilot study was stopped in November of 2017 as the primary care clinic decided to continue and expand the service based on preliminary results. The clinic where the intervention took place is located in a rural Iowa community with a population of 1800 people. This clinic is located in an area with Medically Underserved Area and Medically Underserved Population designations and is the only clinic located in the community. During the 6-month pilot project period, a small population of eligible patients was identified and referred by a primary care provider (PCP) based on current disease status and level of disease control. Patients were identified and recruited by PCPs. Patients were eligible for the intervention if they were 18 years of age or older and had an A1c value of > 7% (8.6 mmol/l). After patients were identified and recruited by PCPs, the pharmacy team providing the service was notified by the PCP and the pharmacist was tasked with contacting the patient for formal introductions. There were four PCPs employed at the clinic at the time of the intervention, and all PCPs participated in the pilot intervention. Patients were followed during the 6-month period by clinical pharmacists and clinical pharmacy interns (i.e., pharmacy students) operating remotely. For this pilot project, the pharmacy team did not seek reimbursement for the services provided; however, potential billable events were recorded.

### Description of clinical service

The remote pharmacy team was comprised of two clinical pharmacists and four clinical pharmacy interns. Clinical pharmacy interns were the first members of the pharmacy team to contact a newly referred patient, and performed additional clinical responsibilities deemed appropriate for their current year in the pharmacy program by supervising pharmacists. Additional responsibilities included but were not limited to mailing disease state management brochures, reconciling medications in the electronic medical record (EMR), and developing care plans for pharmacist approval. Patient encounters were documented in a web-based database designed specifically for use by the remote pharmacy team. The clinical pharmacy team had read-only access to the EMR at the start of the pilot project, but read-write access was later granted at the request of clinic providers. Remote pharmacists provided “in between visit care,” utilizing a Chronic Care Management platform. The remote pharmacists used a semi-structured motivational interviewing [14] approach to engage patients. Motivational interviewing is a patient-engagement process that facilitates behavior change by helping patients explore and resolve their ambivalence or apathy toward change, thus affirming individuals’ autonomy and self-determination while increasing their self-efficacy regarding behavior change. Often, motivational interviewing was required to change patient behavior related to medication adherence, increasing disease control. The pharmacists provided action plans with recommended changes designed to improve disease state control and reduce the gaps in guideline-concordant therapies and preventive care. Further description of intervention activities is included in Table 1.

**Table 1:** Administrative and patient-contact activities of clinical pharmacists

Administrative activities	• Access EMR and collect medical record data to evaluate gaps in therapy for the patient. • Document all patient and provider encounters and time (minutes) used for each activity to determine appropriate billing. • Provide composite tracking and progress reports for all subjects treated by a given physician. • Completed necessary prior authorizations for medication insurance coverage.
Patient-contact activities	• Email, phone, and/or text message the patient every 1–2 weeks × 2 months then monthly to engage patient with self-monitoring. • Use motivational interviewing to conduct monthly follow-up assessment and counseling for medication adherence, side effects, exercise, coronary heart disease (CHD) knowledge, weight management, diet, tobacco use, alcohol use, and associated disease state education. • Assess stages of change for key issues such as exercise, diet, weight management, and tobacco use [15]. • Provide frequent contact with patient to improve preventive health screening. Develop an action plan that addresses gaps in preventive health screening or guideline-concordant therapy, update medication list, and send recommendations for medication changes.

### Outcome measures

For the first study objective, workflow mapping was used to diagram the process and activities of the remote pharmacy team including the clinical pharmacists and clinical pharmacy interns. Patient perceptions of feasibility, appropriateness, helpfulness, and overall experience with the pharmacy team were obtained by a short telephone survey completed by a clinical pharmacy intern who did not interact with the patients during the study. Fifty-two patients were initially identified and referred to the service with 43 patients consenting to participate in the intervention (82.7%). To assess patient and provider perceptions of the intervention, the remote pharmacy team conducted telephone surveys using existing questions that were already established within the healthcare system. Telephone surveys were attempted with all patients and providers who interacted with the pharmacy team during the intervention time period. The telephone surveys were not audio recorded but transcribed during the time of survey by the clinical pharmacy intern. Patients were asked about the extent of pharmacist involvement, including frequency of contact by the pharmacist and perceived benefits of the service. Additionally, patients were asked about potential changes to improve the service moving forward. Patients were asked scaled questions with associated open-ended response options. The survey questions were scaled from 1 (least agreement) to 5 (most agreement) of the given statement. Clinic providers were surveyed to obtain feedback on pilot intervention benefits and potential avenues for improvement or expansion. Providers were asked about perceived value of pharmacist involvement and associated patient care outcomes. Like patients, providers were also asked about opportunities to improve the service.

For the secondary study objectives, A1c values were collected prior to intervention initiation and again upon intervention completion (approximately 6 months). A1c lab values and medication discrepancies were abstracted from the EMR by pharmacy interns. Medication discrepancies were documented as they were identified throughout the duration of the intervention and were evaluated using a previously developed medication discrepancy category list [15]. A subprogram within the pharmacy platform, the Patient Assistance Program (PAP), assisted the pharmacy team when evaluating the patient’s current medication regimens to help determine therapeutically appropriate alternatives for medications at a lower cost. When a therapeutic alternative was identified, the difference in cost and alternative medication were documented. Further, pharmacy interns documented time spent on telephone calls, which was summated at the end of the intervention and reported as mean-per-patient. Lastly, time spent with patients and care plan development were evaluated for their eligibility to be classified as potential billable encounters.

## Data analysis

Means were reported for survey Likert-type responses. Responses to open-ended survey items were categorized. To determine preliminary effects of the pilot intervention, reductions in A1c were calculated and reported. Patients who were identified to receive the pilot intervention were categorized into two groups based on A1c (eAG) levels: > 7% (> 8.6 mmol/l) to < 10% (< 13.4 mmol/l) and ≥ 10% (≥13.4 mmol/l). These groups were determined a priori based on the expertise and experience of the clinical pharmacists and providers, as patients with higher A1c values (≥ 10% (≥ 13.4 mmol/l)) may be subject to varying and intensified therapeutic regimens. Decreases in A1c value were descriptively reported as the average of the individual differences in pre-post A1c values. Medication discrepancies were identified and reported as a percent of all patients experiencing one or more medication discrepancy. Patient medication cost savings were reported as a total annual savings for all patients who participated in the Patient Assistance Program (PAP) provided by the pharmacy team, as well as average savings per patient. Cost-saving interventions included (1) switching patients to medications on the insurance formulary, increasing insurance coverage, and decreasing out-of-pocket posts for the medication and (2) identifying medication assistance programs, which provide medication discounts offered as manufacturer coupons or other discount programs. In addition to cost-saving interventions, the PAP involved completing prior authorizations for patients which is the process of obtaining insurance approval to initiate therapies. While not directly associated with patient cost savings, obtaining approval allowed for appropriate therapy to be initiated due to insurance coverage of medications. There was no billing for services in this pilot intervention. However, a projected ROI was calculated based on number of potentially billable CCM interventions performed compared to cost of the pharmacy service. The cost of the service was calculated based on hourly rates of the pharmacist team performing the intervention. As the clinical pharmacy team has been established for multiple years, institutional and organizational funding support the overhead and facility costs of the service. These costs were not included, as they were not costs the rural clinic was directly responsible. ROI was reported based exclusively on the potential costs the pharmacy would charge the clinic for their service subtracted from the revenue the clinical pharmacist team generated for the rural clinic.

## Results

### Workflow process

The workflow process for recruiting patients and subsequent monitoring was adapted to meet the needs of the rural clinic. PCPs were responsible for identifying eligible patients and notifying the remote pharmacists or clinical pharmacy interns. Pharmacists were initially granted read-only privileges in the clinic EHR, but obtained read-write capabilities upon PCP recommendation. PCPs were also responsible for reviewing pharmacist recommendations and responding to these recommendations within three business days. PCPs were then asked to insert the pharmacist communications into the patient’s medical record to serve as patient progress notes. Lastly, PCPs were asked to update the pharmacy team with any changes in medication regimen following clinic visits.

The pharmacy service was integrated into the on-site primary care team with frequent two-way communication with providers and their patients. After PCP patient referral, pharmacy personnel performed in-depth chart review in the EMR for every patient who agreed to participate in the program. Pharmacists identified therapy enhancements and cost-saving opportunities related to medication use and contacted patients by phone.

**Fig. 1:**
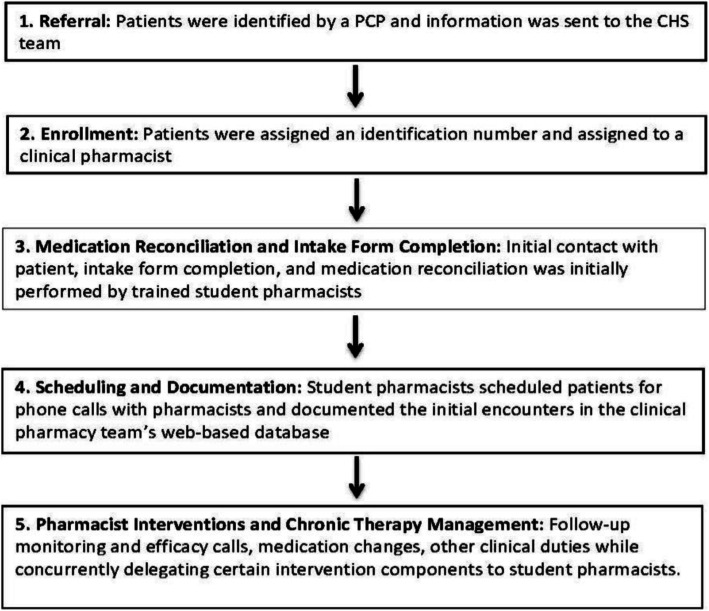
Example of pharmacy workflow

Lastly, the rural clinic staff was responsible for ordering necessary labs when recommended by pharmacists and approved by the PCP, updating EMR medication lists when discrepancies were identified by the pharmacists, and electronically submitting medication refills per clinic protocol when notified by the pharmacist and approved by the provider. All communication with the PCP was performed via secure email messaging, and communication with clinic staff was performed with secure email messaging as well as phone conversation. General workflow is further depicted in Fig. 1.

### Survey data

PCPs identified and referred 52 patients to the service, of which 43 agreed to participate in the intervention. Twelve patients completed telephone surveys for a response rate of 28% (*n* = 12/43), and all four providers involved in the intervention completed telephone surveys for a response rate of 100% (*n* = 4/4). Overall, patients found the pharmacists to be helpful and felt they were being contacted at an appropriate frequency. Further, patients agreed that working with the pharmacy team reduced medication costs. Scaled responses are presented in Table 2. Patients reported that improving knowledge, adherence, and diabetes control were major reasons for perceived helpfulness of the service. Patients also wanted to have continued access to the pharmacist after the pilot project ended, and some patients were willing to pay an out-of-pocket fee to be able to continue the service. Patient responses to survey questions are included in Table 3.

**Table 2:** Scaled patient responses from telephone surveys

Scaled question	Average response value*
The pharmacist was helpful	4.75
The pharmacist reduced medication costs	3.16
Frequency of pharmacist calls was appropriate	4.3

*1 = least agreement, 5 = most agreement

**Table 3:** Patient responses to open-ended questions

Survey question	Open-ended response
Why did you agree to visit with a pharmacist?	“Doctor recommendation.”
	“Wanted different opinion.”
	“Wanted more information about medications.”
	“Uncontrolled chronic conditions.”
What was your reasoning for your rating of the pharmacist call helpfulness?	“Provided more information/improved [my] understanding.”
	“Reminders helped increase adherence and home monitoring.”
	“Pharmacist was a good listener, expressed empathy and compassion.”
What was your reasoning for your rating of pharmacist reducing medication costs?	“Pharmacists helped lower [my] medication costs.”
What was your reasoning for your rating of talking with a pharmacist was beneficial to my health?	“Pharmacist provided detailed explanations about [my] meds.”
	“Pharmacists improved BP, A1c.”
	“Increased awareness about health conditions.”
	“Helped understand importance of daily monitoring.”
	“Pharmacist was accessible to answer [my] questions.”
	“Pharmacist worked to find best solution for [my] specific needs.”
What did you like about having the pharmacist talk to you?	“Pharmacist available to answer [my] questions.”
	“Pharmacist had unique ideas to improve health.”
	“Pharmacist helped with lifestyle behaviors (e.g. meal modification and alcohol consumption).”
	“Clarified areas of confusion.”
	“Increased accessibility to healthcare professional.”
If the service is continued, what can be done to improve it?	“Nothing.”
	“Be clearer about when service/communication is ending.”
	“No negative experiences.”
	“Lower cost of medications.”
Overall comments	“Would like pharmacist to be available for questions as they arise.”
	“Very thankful and appreciative of the service.”
	“Frustrated by ceased communication.”
	“Would like to talk less to pharmacists now that things are under control.”
	“Pharmacist cared, asked question, gave information, and tried to get me on the right track and right medications.”
	“Provided additional information and improved understanding of meds and disease state.”
	“Calls helped increase adherence and home monitoring.”
	“Pharmacist was available when clinic wasn’t open.”
	“Pharmacist was a good listener, expressed empathy and compassion.”

Provider responses are included in Table 4. Provider and clinic staff encouraged continuation of the program and also had suggestions for workflow and process improvement. Additionally, provider and clinic staff experiences were generally positive, with a number of perceived program benefits identified. Clinic providers reported that the pharmacy intervention did not create extra work and, instead, freed up time for other activities. Clinic staff noted that pharmacist completion of prior authorizations was very helpful, especially since it was estimated by clinic staff that 20 diabetes-related prior authorizations/month needed to be completed. Clinic staff reported receiving positive feedback from patients regarding assistance with medication cost savings, understanding what their medications were for, and having another healthcare member involved in their care.

**Table 4:** Provider feedback to overall program experience

Provider experience comments	
“Pharmacists did reminder calls for labs and needing to schedule appointments.”	
“Minimum of 30 minutes spent on each prior authorization, sometimes up to an hour.”	
“Would allow me to see more patients since pharmacist would collect information prior to the clinic visit.”	
“Helped improve the care of my patients by following up with them before clinic visits.”	
“Liked having them watch out for drug interactions and renal dosing adjustments.”	
“Med errors are often reason for hospital or ED visit. Working with pharmacists helps prevent that.”	
“Would prefer pharmacists document directly into EMR.”	
“Figure out what could be billed through insurance so patients can keep using the service.”	
“Pharmacists tailored service for patient’s needs.”	

### Decreases in A1c

During the pilot period, there was an A1c decrease in both groups, based on the average individual difference in pre-post intervention A1c values. For patients with uncontrolled diabetes with an A1c of greater than or equal to 10% (an eAG of 13.3 mmol/l), the average individual difference in pre-post intervention A1c values was 2.133%. For patients with uncontrolled diabetes and an A1c ranging from 7 to 9.9% (an eAG ranging from 8.6 to 13.33 mmol/l), the average individual difference in pre-post intervention A1c values was 2.0%.

### Medication discrepancies

Pharmacists identified at least one medication discrepancy in 19 of the 43 patients (44%). The total number of discrepancies ranged from 1 to 5 per patient, with the most common types of discrepancies being medications prescribed by outside provider not documented in the primary care provider’s EMR (*n* = 6), inactive medications still active in the primary care provider’s EMR (*n* = 5), routine over-the-counter (OTC) medications not included in the primary care provider’s EMR/patient taking medications not on the EMR (*n* = 5), and medication samples not documented in the primary care provider’s EMR (*n* = 2).

### Medication cost savings

When evaluating lower-cost medication alternatives, the PAP identified five individuals who could benefit from this subprogram. Collectively, these patients experienced a total annual savings of $3,360 USD from the utilization of the pharmacy PAP service, resulting in an average savings of $672 USD per patient. Cost savings were realized when pharmacy staff identified lower-cost therapeutic alternatives based on individual insurance coverage. These were often chronic medications, which were changed based on pharmacist recommendation. Medication classes where lower-cost alternatives were identified included antidiabetics and oral anticoagulants.

### Potential return on investment

Time spent on consultation, reimbursement per consultation, and number of billable events are listed in Table 5. Overall, the pharmacy team spent 9978 min (166.3 h) performing clinical pharmacy services, an average of 243.4 min performing clinical services/patient over the course of the 6-month intervention. Patients received varying amounts of contact with clinical pharmacy personal, with a range of 104 to 556 min. The pharmacy team would have generated $3986 in revenue through CCM reimbursement. The potential ROI had the pharmacy team submitted for Medicare reimbursement was 1.4:1.

**Table 5:** Potential return on investment (ROI) based on pharmacy billable services

Time spent/consultation (minutes)	Chronic Care Management code	Reimbursement*	No. of billable events	Potential revenue
20	99490	$43	14	$602
60	99497	$94	12	$1128
90	99487 + 99489	$141	16	$2256
			**Reimbursement**	**$3986**
			**Cost of service**	**$2846**
			**Potential net profit**	**$1140**
			**ROI**	**1.4:1**

*Reimbursement amounts based on Iowa Medicare billing in 2017

## Discussion

The remote pharmacy service model was feasible to integrate into a rural clinic setting, with positive outcomes identified during the pilot project period. Based on patient and provider telephone survey responses, the pharmacy service and clinical pharmacists were well received. Provider feedback was generally positive, with constructive criticism on program improvement opportunities provided. Providers preferred for the pharmacists to have read-write EMR access to further optimize workflow efficiency.

We achieved the primary objective of this pilot study and demonstrated feasibility and acceptability of recruitment and retention of patients into a pharmacist-led telehealth intervention for diabetes management. The workflow process was appropriate for the rural clinic setting, and utilization of pharmacy interns provided an additional avenue of cost containment due to relatively lower salary requirements for interns. Pharmacy interns were able to perform clinical activities with appropriate training and under supervision of the pharmacist. For changes in A1c, the intervention appeared to contribute to A1c reduction in the recruited patient population. To determine true intervention effectiveness, a larger, adequately powered pragmatic study would need to be performed. According to clinic staff, an A1c value of 8.0% is the quality metric target set by a number of insurance payors that results in increased or incentivized reimbursement for quality performance. Given the short duration and nature of this pilot and feasibility study, these metrics were not met at study endpoint; however, it is reasonable to assume given the downward trend in A1c values that some patients would likely meet this threshold. Meeting this A1c threshold would result in increased payment based on quality. Overall, the intervention resulted in better diabetes control, identification of medication discrepancies, and considerable cost savings for a small subset of the pilot intervention population.

### Potential return on investment

The Centers for Medicare & Medicaid Services (CMS) recognizes that Chronic Care Management (CCM) is a critical component of primary care that promotes better health and reduces overall healthcare costs [1]. CCM services are typically non-face-to-face services provided to patients who have two or more chronic conditions expected to last at least 12 months. These services help patients with chronic conditions establish comprehensive care plans with appropriate clinician oversight. CCM services have been billable since 2015, and while readily available to physicians and other non-physician practitioners, pharmacists are not specifically mentioned as a practitioner eligible to bill for CCM services. However, CCM services that are not provided personally by the billing practitioner may be provided by clinical staff, provided the clinical staff are under contract with the billing practitioner and the services fall within the clinic staff scope of practice [1].

Outside of the ROI calculation based on billable patient encounters, there are a number of additional return opportunities based on the way reimbursement and workflow are currently structured in the healthcare system. First, in a constantly changing atmosphere of healthcare quality, additional revenue may be generated from meeting or exceeding established quality benchmarks or targets set by individual payors. In shared savings payor contracts, opportunity exists to receive enhanced incentive payments from insurance payors for reducing the total cost of care. A pharmacist-led intervention focused on switching patients to lower-cost alternative therapies, selecting payor preferred drug therapies, improving chronic disease management, and reducing potential adverse drug events can reduce the total cost of care. Lastly, pharmacy provision of these services has the potential to free up clinic staff for other tasks that are essential to providing quality, timely healthcare services to patients. The pharmacy service was reported to increase staff time available for other services and tasks by assuming the responsibility of performing prior authorizations for medications on patients enrolled in the pilot. Additionally, the pharmacists queued prescription refills for patients and providing appropriate and timely follow-up on medication efficacy and side effects which was previously a responsibility of clinic staff. As diabetes treatment continues to become more complex with higher risk for drug-drug and drug-disease interactions, pharmacist involvement may prove increasingly valuable.

The pharmacy service is currently exploring the opportunity for Transitional Care Management (TCM) billing, where pharmacy will perform non-face-to-face phone call within two business days of hospital discharge, which could be billed at an estimated rate of $168 or $238 per encounter depending on complexity of the patient case. While these phone calls are only a small component of the TCM billing requirements, they are a component with considerable potential to improve transitions of care. Other opportunities to expand the service exist including evaluating unnecessary medication use (e.g., proton pump inhibitors without indication), appropriate anticoagulation selection, anticoagulation management, and tobacco cessation. The use of pharmacy student interns for initial patient encounters and intake form collection resulted in a cost of $15/hour to perform these tasks. The projected cost for pharmacist-performed initial encounters and intake form collection was $1540, or $50/hour for 30.8 h of service.

### Limitations

The results of the pilot project must be considered in light of limitations. This pilot project was limited in the beginning due to the pharmacy team having read-only access to the EMR, preventing them from making real-time therapy recommendations to providers. Further, the duration of the project was short since the main objective of this project was designed to assess workflow as well as patient and provider satisfaction with and perceived value of the service. The secondary measure, change in A1c, was trending downward but would likely need a longer project period to examine sustained effectiveness of the service. Analysis was not performed to detect statistical significance of A1c decreases in pre-post groups, which should be performed upon completion of a larger, randomized study. The patient survey response rate was low (28%) making the patient experience difficult to generalize. Additionally, the utilization of pharmacy students within clinical pharmacy workflow may be non-applicable outside of services housed in an academic institution. Lastly, medication adherence and other patient-specific changes were not evaluated in this pilot study despite pharmacists being actively involved in the medication utilization process. Medication refills and adherence could be evaluated in future studies.

## Conclusions

Overall, the use of centralized, remote pharmacy services in the rural clinic setting was well received and feasible. Based on initial results from a small sample size, the pharmacy team was effective at assisting with management of diabetes, identifying and reducing medication discrepancies, and helping individuals afford their medications. Additionally, clinic providers and staff reported having more time to perform other duties in the clinic. Further research should be done to evaluate the effectiveness of interventions in additional rural clinic settings, additional disease states, and with expanded services based on patient and physician testimony.

## Data Availability

The datasets used during the current study are available from the corresponding author on reasonable request.

## Abbreviations

ROI: Return on investment
TCM: Transitional Care Management
CCM: Chronic Care Management
EMR: Electronic medical record
PAP: Patient Assistance Program
PCP: Primary care provider
